# Supplementary material for: Early detection and staging of chronic liver diseases with a protein MRI contrast agent
Source: Nat Commun. 2019 Oct 29;10:4777. doi: 10.1038/s41467-019-11984-2 (PMC6820552; doi:10.1038/s41467-019-11984-2)
Supplement: Supplementary file 1 — Supplementary Information [file 41467_2019_11984_MOESM1_ESM.pdf]

Early Detection and Staging of Chronic Liver Diseases with a Protein MRI Contrast Agent

Salarian et al.

Supplementary Figure 1

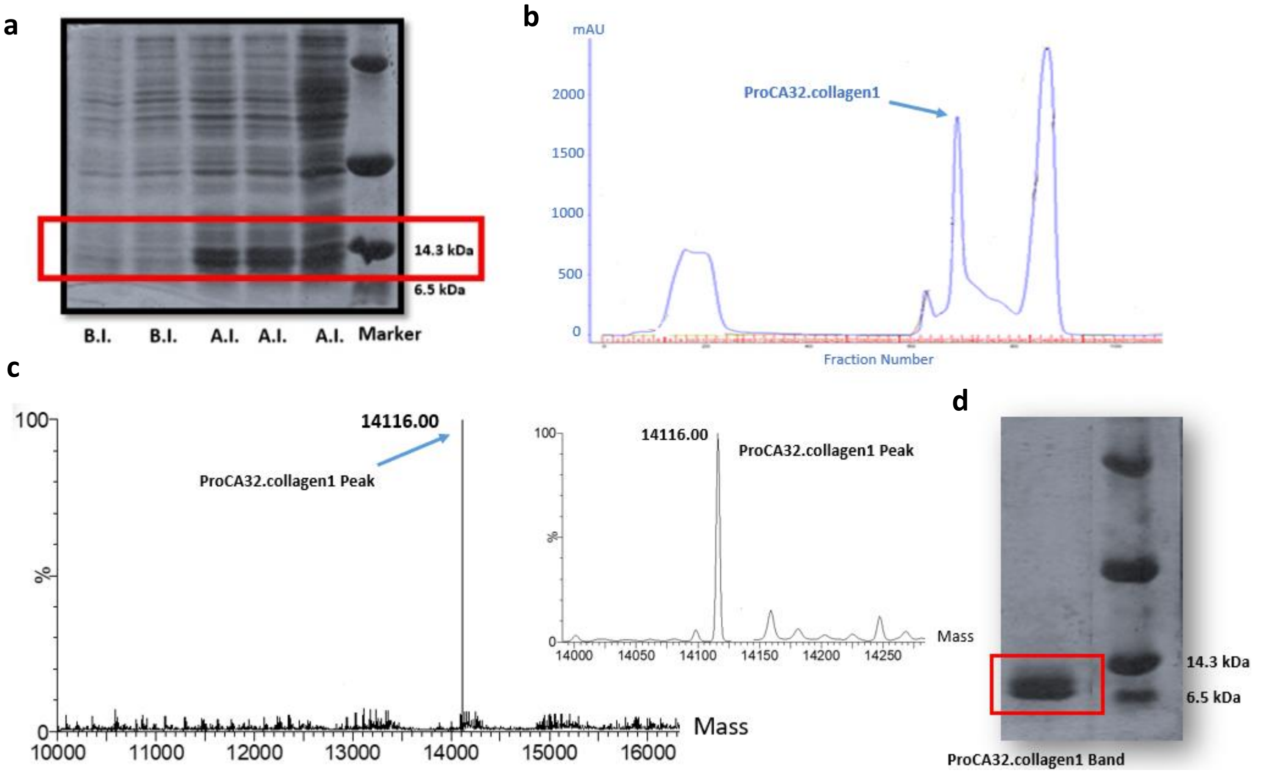

Supplementary Figure 1: Expression and purification of ProCA32.collagen1. **a:** SDS-page results with Coomassie brilliant blue staining demonstrates the expression of ProCA32.collagen1 (BI: Before induction of IPTG; AI: After induction of IPTG). **b:** Ion exchange chromatogram shows purified ProCA32.collagen1 band. **c:** Electrospray ionization mass spectrometry of ProCA32.collagen1 with calculated molecular weight. **d:** SDS-page results with Coomassie brilliant blue staining demonstrates the purified ProCA32.collagen1 band close to 14.3 kDa band.

Supplementary Figure 2

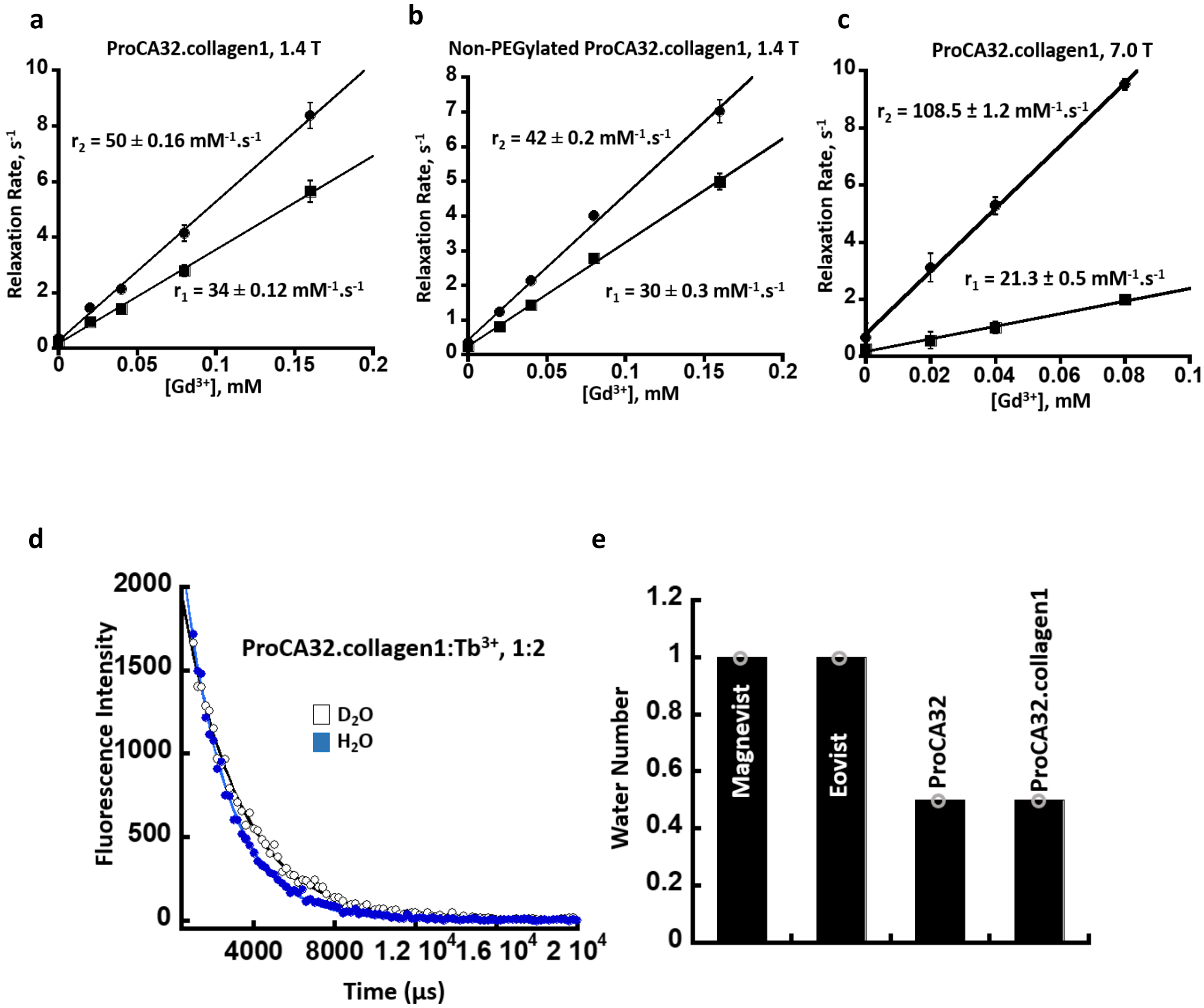

Supplementary Figure 2: Relaxivity and water number measurements of ProCA32.collagen1**. a:** Relaxivity of ProCA32.collagen1 at 1.4 T, 37 °C. Changes in r_1 and r_2 relaxation rates were plotted over various concentrations of [Gd^{3+}]. **b:** Relaxivity of non-PEGylated ProCA32collagen1 at 1.4 T, 37 °C. **c:** Relaxivity (r_1 and r_2) of ProCA32.collagen1 at 7.0 T, 37 °C. **d:** The luminescence decay of Tb^{3+} in H_2O or D_2O in solution of ProCA32.collagen1. Tb^{3+} and ProCA32.collagen1 were mixed with 2 to 1 ratio. **e:** Water number of Magnevist, Eovist, ProCA32, and ProCA32.collagen1. Error bars indicate standard deviations of three separate measurements.

Supplementary Figure 3

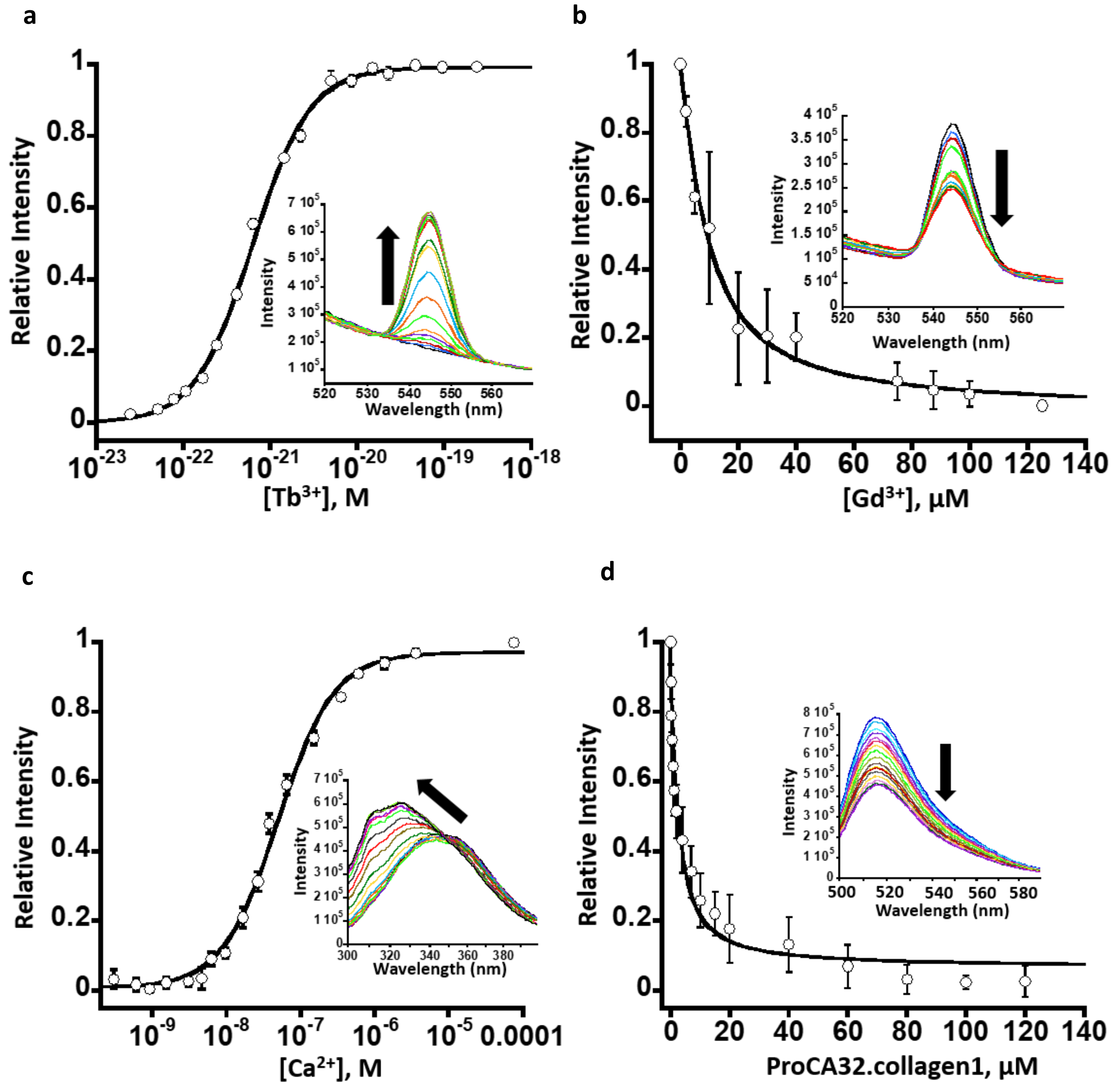

Supplementary Figure 3: Determination of metal binding affinities of ProCA32.collagen1. **a:** Determination of Tb^{3+} binding affinity of ProCA32.collagen1 using Tb-DTPA chelator buffer system. The interaction between Tb^{3+} and ProCA32.colagen1 was quantified by fluorescence intensity increase. **b:** Gd^{3+} binding affinity was calculated using competition assay. The fluorescence intensity decreases due to luminescence resonance energy transfer between Trp in

ProCA32.colagen1 and bounded Tb^{3+} was quantified when Gd^{3+} competed Tb^{3+} out of the metal binding pocket. **c:** Determined Ca^{2+} affinity to ProCA32.collagen1 using the Ca^{2+}-EGTA buffer system. The interaction between Ca^{2+} and ProCA32.colagen1 was monitored by the Trp fluorescence intensity increase by increasing Ca^{2+} concentration. **d:** Zn^{2+} binding affinity was determined using Fluozin-1 competition assay in which ProCA32.collagen1 is competing with Fluozin-1 for Zn^{2+}. Error bars indicate standard deviations of three separate measurements.

Supplementary Figure 4

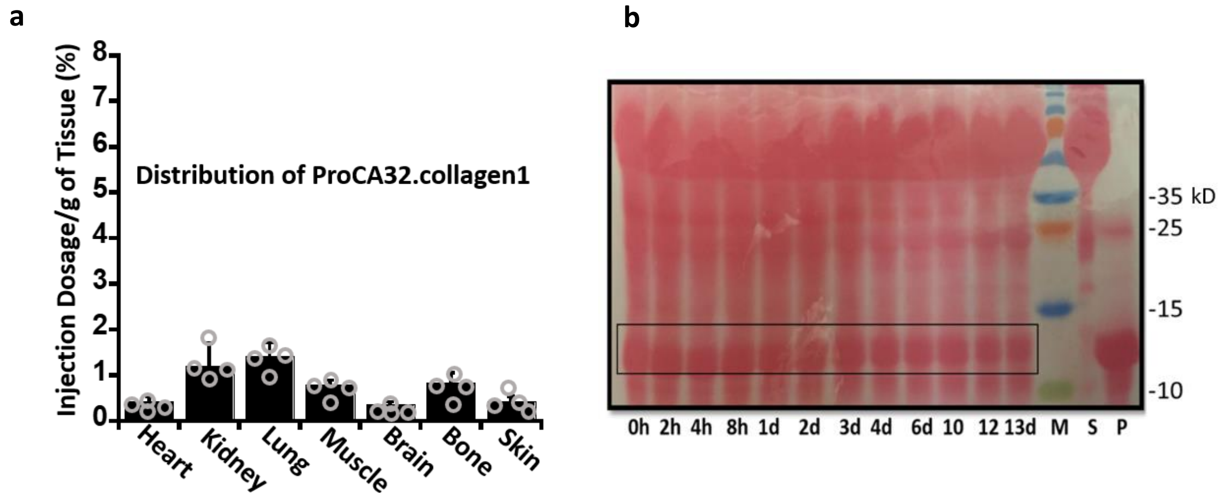

Supplementary Figure 4: Biodistribution and serum stability analysis of ProCA32.collagen1. **a:** Shows percent injection dosage of ProCA32.collagen1 (0.02 mmol/kg) based on [Gd^{3+}], in other organs. ProCA32.collagen1 does not have brain deposit possibly due to lack of penetration of the blood brain barrier (BBB). **b:** ProCA32.collagen1 is stable after incubation in serum at 37 °C for up to 12 days as shown by Ponceau S staining, S: Serum, P: Protein. Data are represented as mean ± SD (n=4).

Supplementary Figure 5

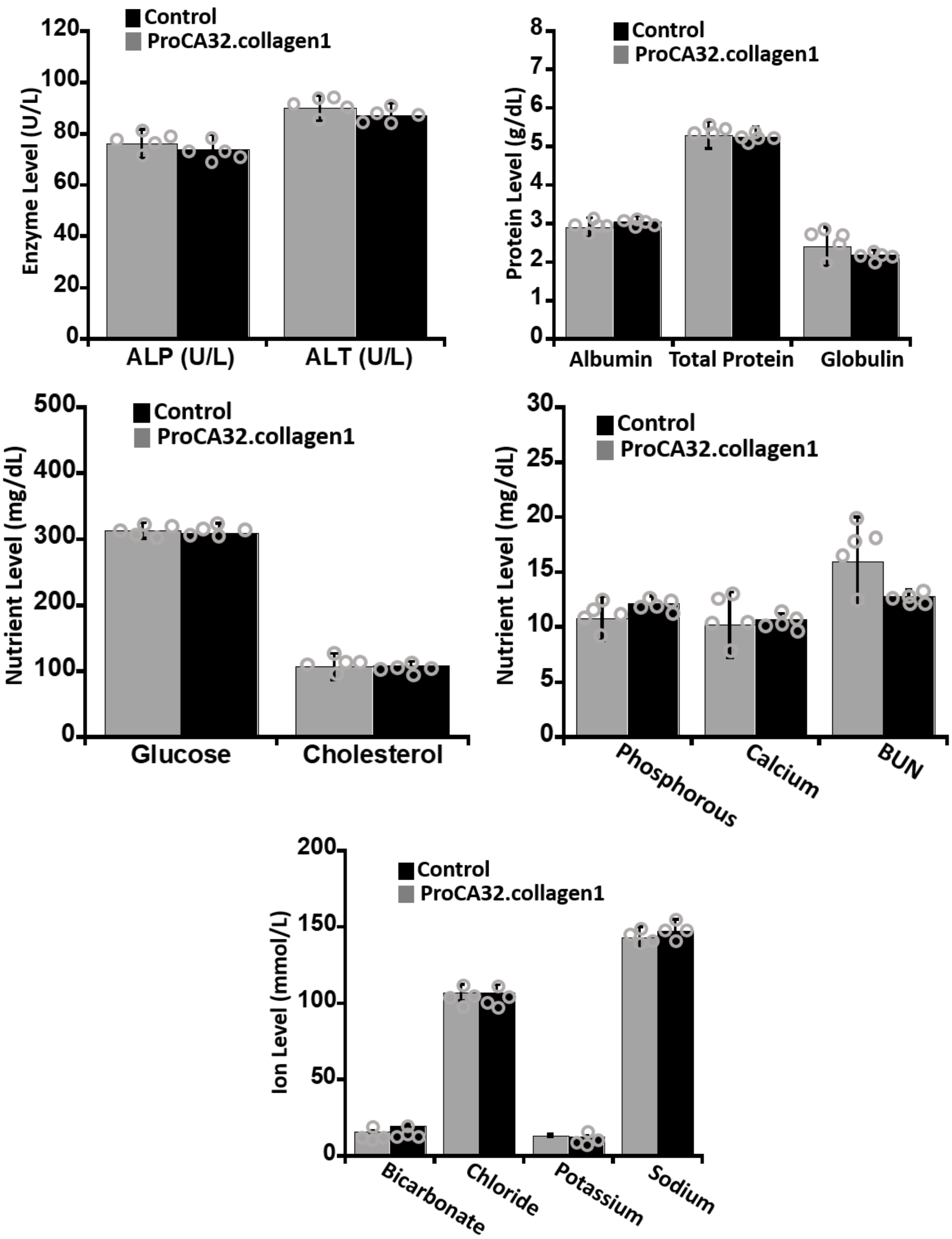

Supplementary Figure 5: Clinical chemistry studies of ProCA32.collagen1. Clinical chemistry tests after injection of 0.02 mmol/kg, ProCA32.collagen1. Each organ was collected, and 0.2 g was digested with concentrated HNO_3 then diluted with DD water and analyzed by ICP-OES. The mice clinical chemistry profiles were collected by analyzing mice serum samples 5 days after injection of saline (gray, n = 5), or 0.02 mmol/kg ProCA32.collagen1 (black, n = 5). Data are expressed as mean ± SD.

Supplementary Figure 6

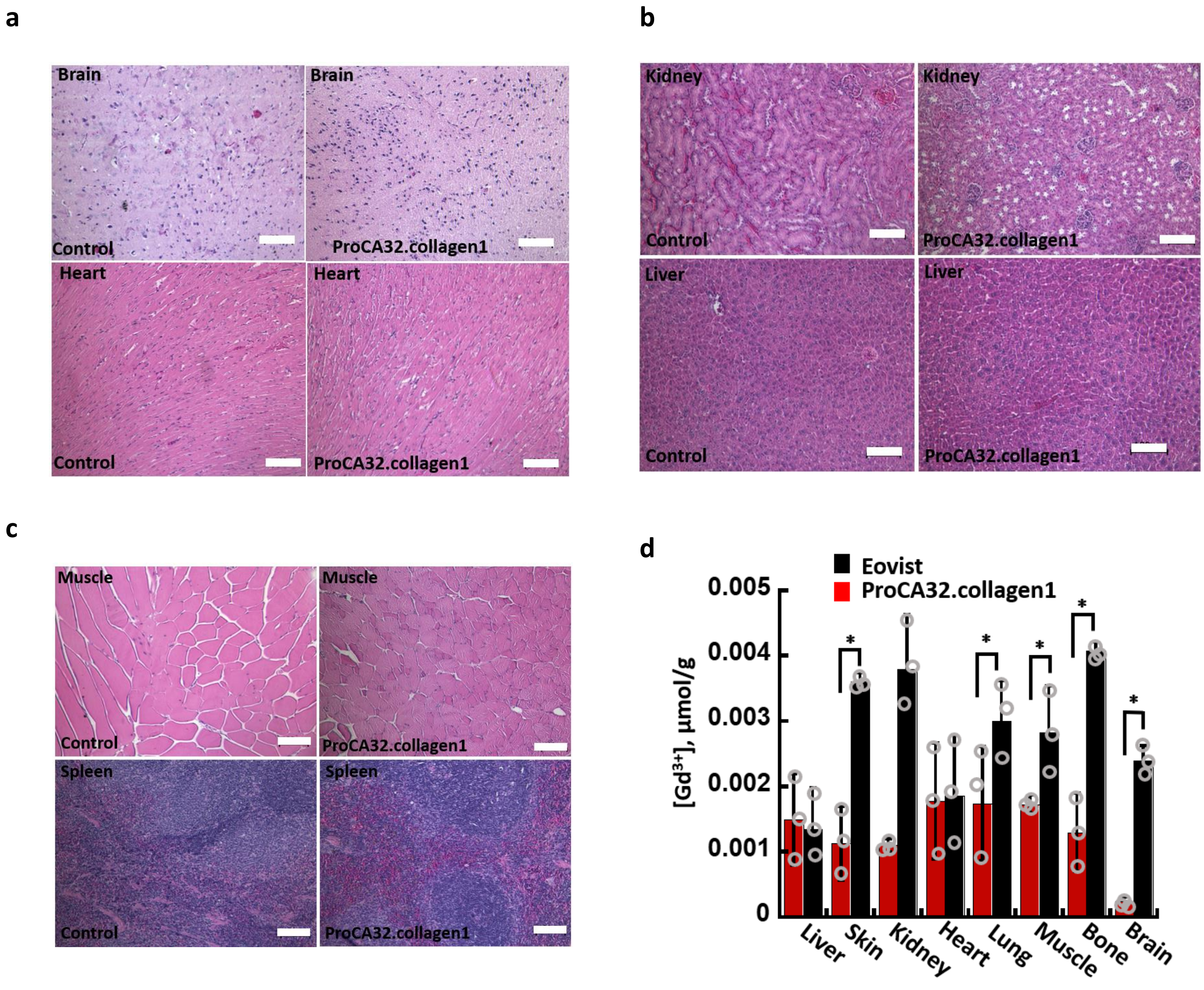

Supplementary Figure 6: Tissue damage assessment of ProCA32.collagen1. **a, b, c:** ProCA32.collagen1 (0.02 mmol/kg) or saline (control) were injected to CD-1 mice. H&E staining was used to evaluate the damage of the ProCA32.collagen1 to these tissues after injection. An experienced pathologist was blinded to the groups of H&E staining. ProCA32.collagen1 shows no signs of tissue damage due to Gd^{3+} toxicity in organs studied, including liver, kidney, muscle, spleen, brain, and heart. The images are representative of three independent experiments. **d:** Long term organ Gd^{3+} deposition demonstrates the biodistribution of ProCA32.collagen1 and Eovist 14 days after injection in mice in different organs using ICP-OES. After injection of Eovist, different organs such as brain, skin and bone have high accumulations of Gd^{3+}. scale bar, 100 μm; $*P < 0.05$, student's *t*-test; Data are represented as mean ± SD (n=3).

Supplementary Figure 7

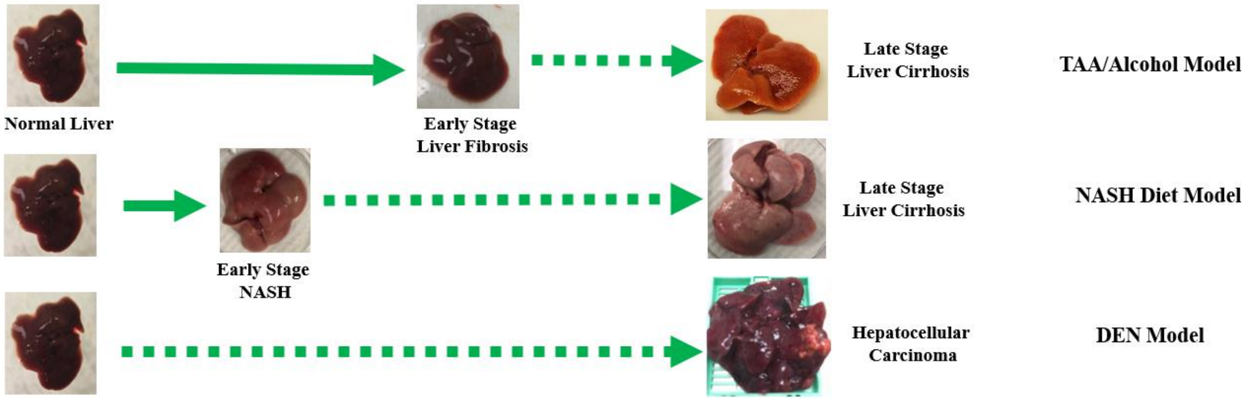

Supplementary Figure 7: Animal models of liver fibrosis and NASH. TAA/alcohol-, DEN- and NASH diet-induced liver fibrosis were developed according to the procedure described in Methods section.

Supplementary Figure 8

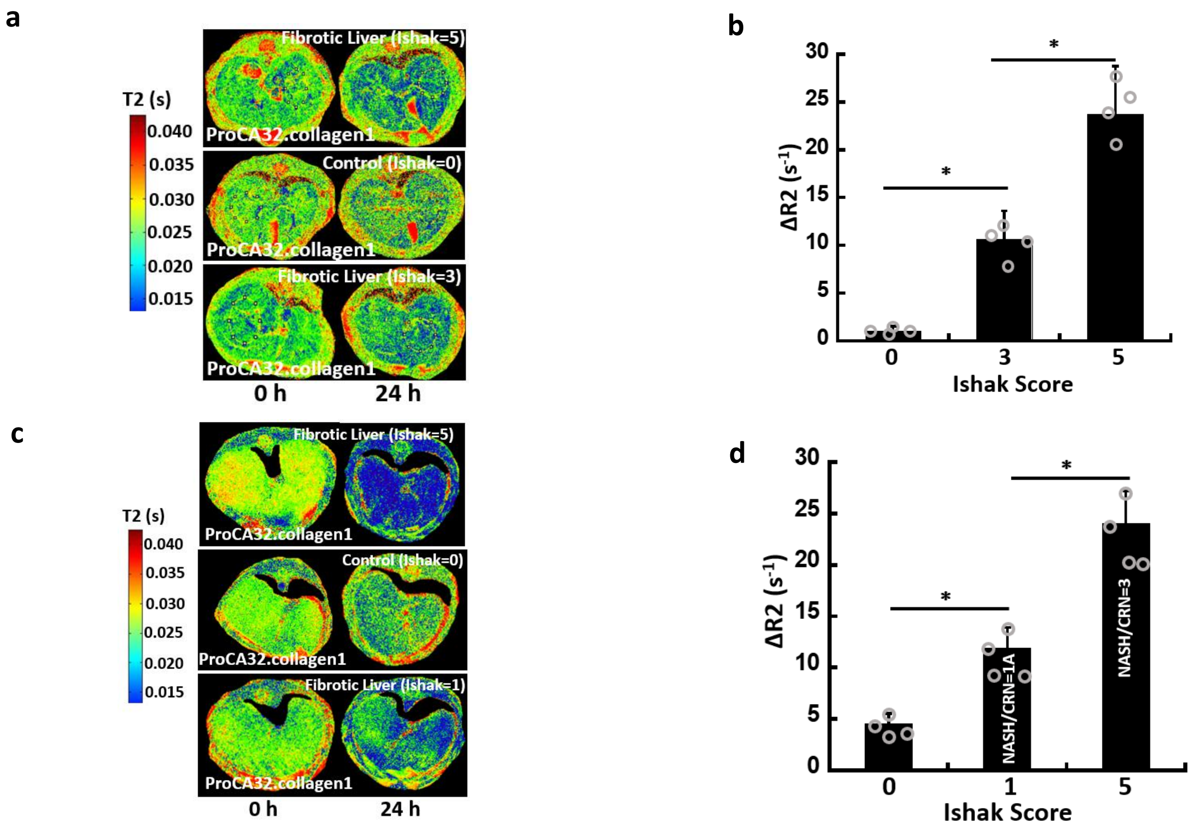

Supplementary Figure 8: Early stage detection of liver fibrosis and NASH induced by TAA/Alcohol and NASH diet with T2 mapping with ProCA32.collagen1. **a:** T2 maps of early-stage (Ishak 3 of 6), normal (Ishak stage 0 of 6) and late-stage (Ishak stage 5 of 6) liver fibrosis before and 24 h after injection of ProCA32.collagen1 in TAA/Alcohol model. **b:** ProCA32.collagen1 can distinguish early-stage liver fibrosis from normal liver and late-stage liver fibrosis with ΔR2. **c:** T2 maps of early-stage NASH liver (Ishak stage 1 of 6 or Mild-1A in NASH CRN system), late-stage liver fibrosis (Ishak stage 5 of 6 or 3 in CRN system), and normal liver (Ishak stage 0 of 6) before and 24 h after injection of ProCA32.collagen1. **d:** ProCA32.collagen1 can distinguish early-stage NASH from normal liver and late-stage liver fibrosis with ΔR2. *P < 0.05, student's *t*-test; Data are represented as mean ± SD (n=4).

Supplementary Figure 9

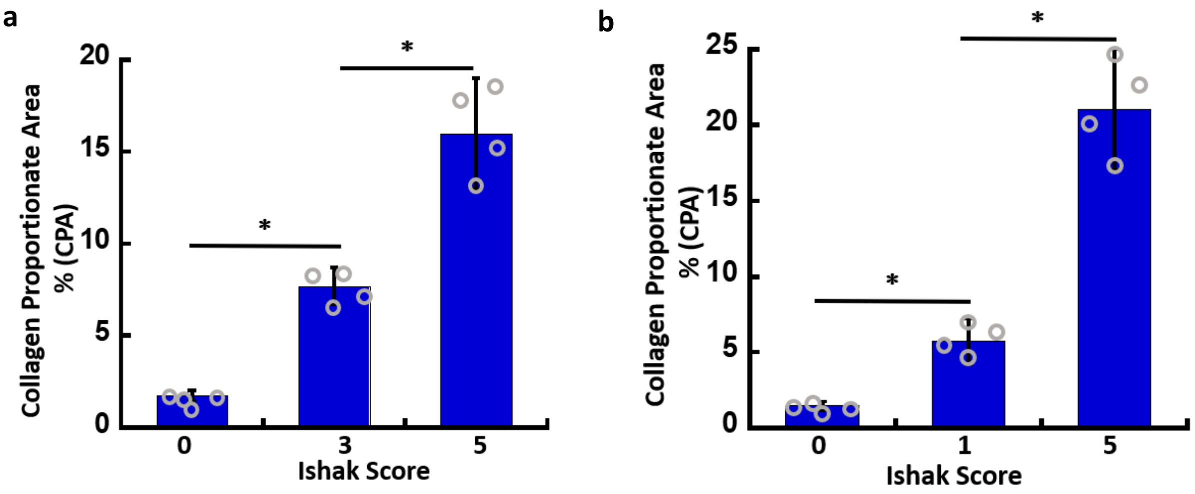

Supplementary Figure 9: Collagen proportionate area (CPA) analysis of mouse liver tissues. **a:** CPA percentage calculation of mouse liver tissues on Sirius red stained slides in TAA/Alcohol mouse model. **b:** CPA percentage calculation of mouse liver tissues on Sirius red stained slides in NASH diet mouse model in three different stages. *P < 0.05, student's *t*-test; Data are represented as mean ± SD (n=4).

Supplementary Figure 10

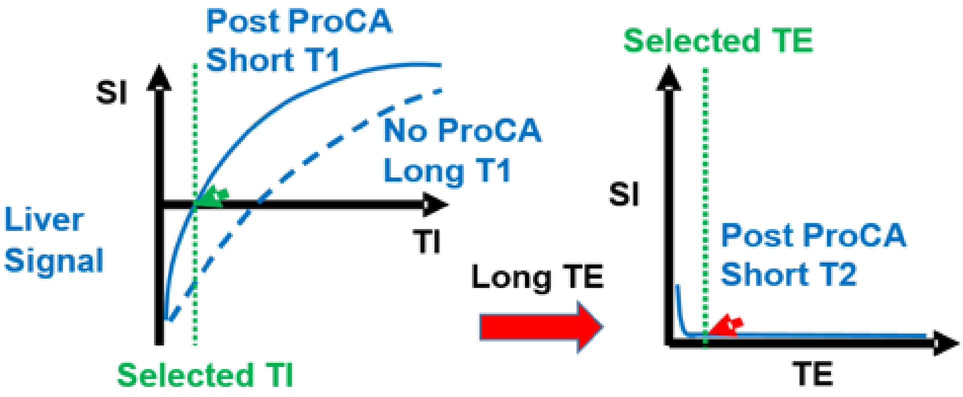

Supplementary Figure 10: Schematic demonstration of application of short T1 inversion recovery with long TE methodology with ProCA32.collagen1. ProCA32.collagen1 has high accumulation in the liver. As a result, liver has shortened T1, first, inversion time was used to suppress liver signal. Second, since ProCA32.collagen1 also has short T2, a long TE was used to further suppress the liver signal.

Supplementary Figure 11

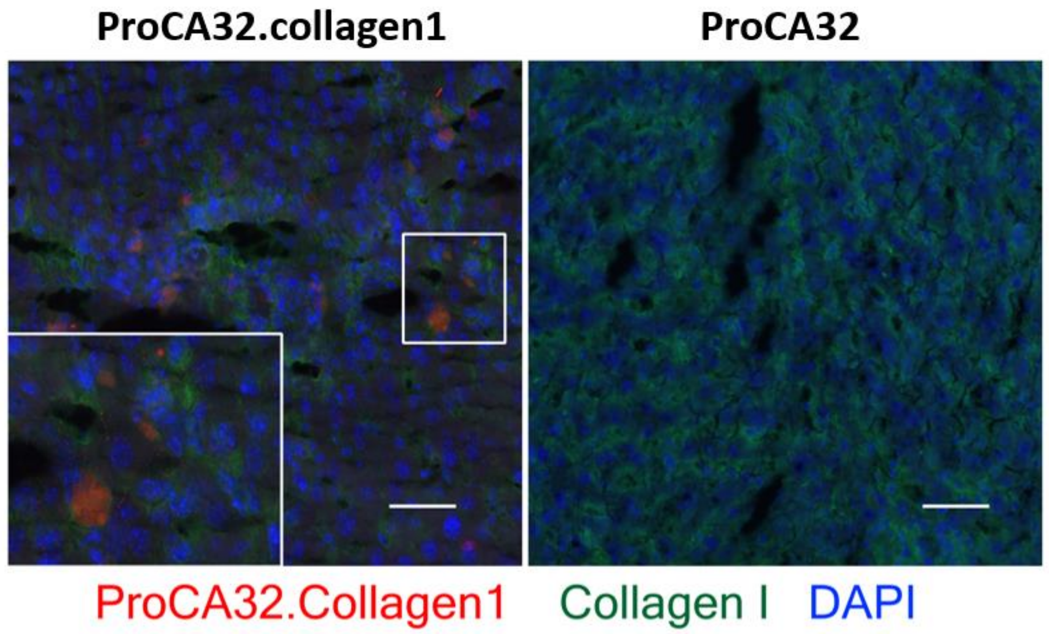

Supplementary Figure 11: Immunofluorescence staining of ProCA32.collagen1, ProCA32 and collagen type I in fibrotic liver tissues. Fibrotic liver injected with ProCA32.collagen1 demonstrates that ProCA32.collagen1 (red) can target collagen type I (green) in liver, however, ProCA32 did not show any targeting capability as it cannot be detected by immunofluorescence imaging in fibrotic liver, scale bar, 100 µm.

Supplementary Figure 12

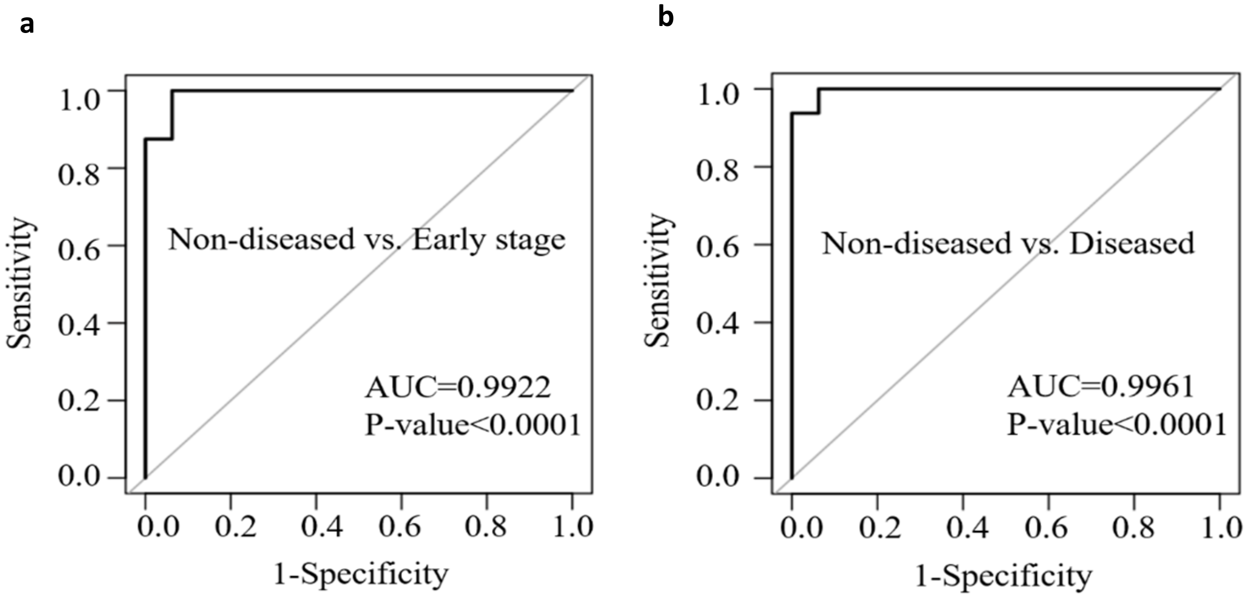

Supplementary Figure 12: Statistical analysis of TAA/Alcohol-induced liver fibrosis detection with ProCA32.collagen1. **a:** Receiver Operating Characteristic (ROC) analysis demonstrated that ΔR1 could detect early-stage fibrosis and distinguish CPA $\leq$ 1.75% mice from CPA >1.75% (AUROC = 0.9922, P < 0.0001), using 24-hour time point ΔR1 data (predicting non-diseased vs. early stage). **b:** ROC analysis shows that using both ΔR1 3 and 24-hour time points, ProCA32.collagen1 can distinguish diseased (early- and late-stage liver fibrosis) from normal liver (AUROC = 0.9961, P < 0.0001, unpaired two-tailed student's t-test, n=4).

Supplementary Figure 13

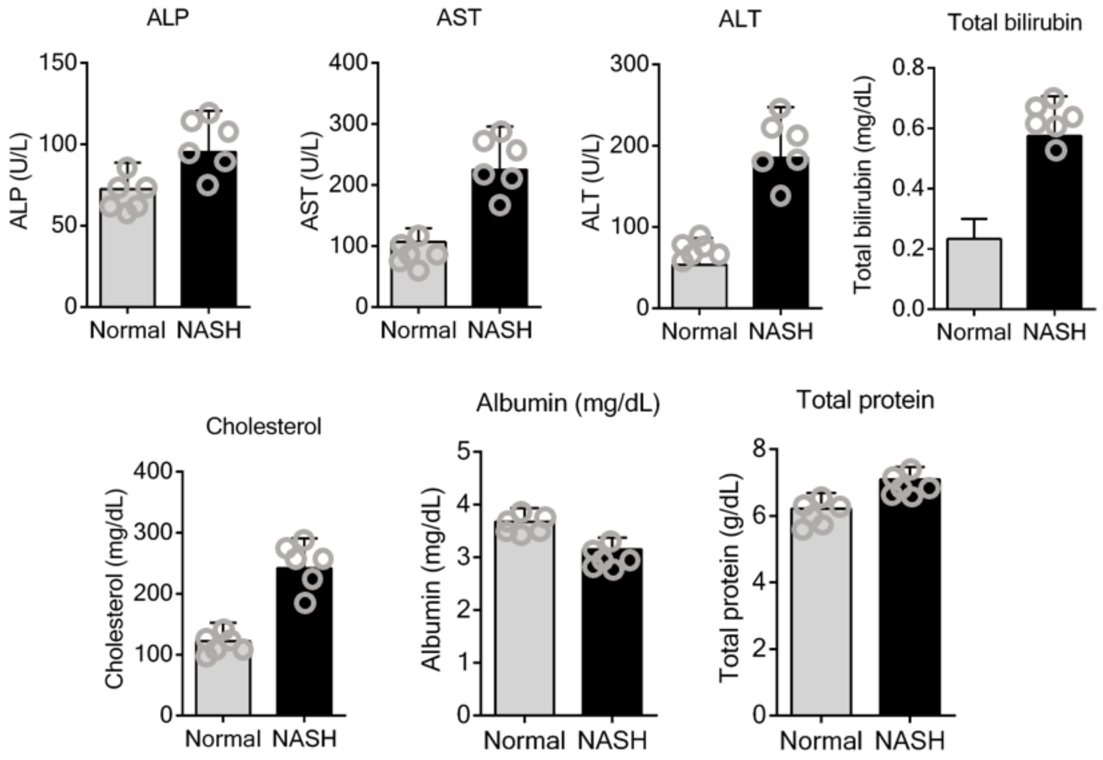

Supplementary Figure 13: Levels of serum markers in blood circulation of normal mice compared to LivKO mice treated with NASH-diet. The samples were analyzed via commercial service (CPath). Error bars indicate standard deviations of six randomly selected mice from each group.

Supplementary Figure 14

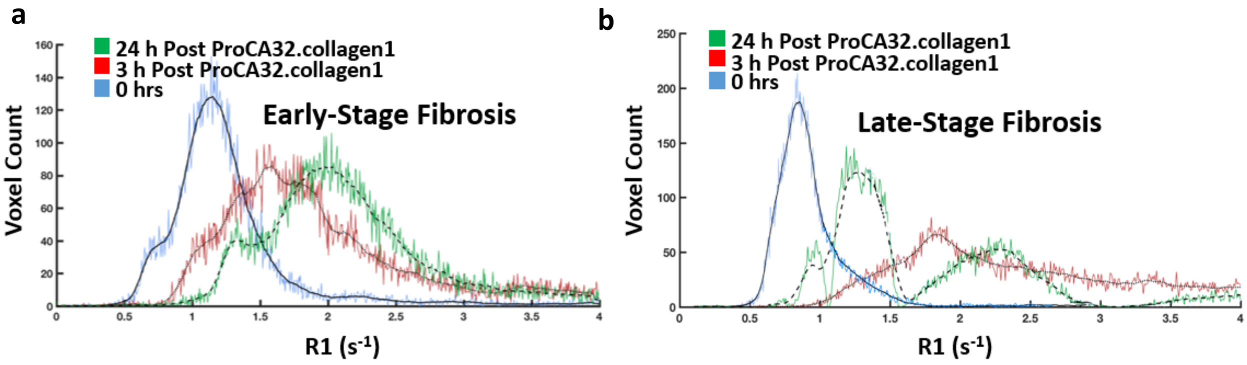

Supplementary Figure 14: Histogram analysis of liver R1 values in TAA/Alcohol mouse model. **a:** Histogram analysis of early-stage liver fibrosis (Ishak stage 3 of 6), pre (blue), 3 h (red) and 24 h (green) post injection of ProCA32.collagen1. **b:** Histogram analysis of late-stage liver fibrosis (Ishak stage 5 of 6), pre (blue), 3 h (red) and 24 h (green) post-injection of ProCA32.collagen1.

Supplementary Figure 15

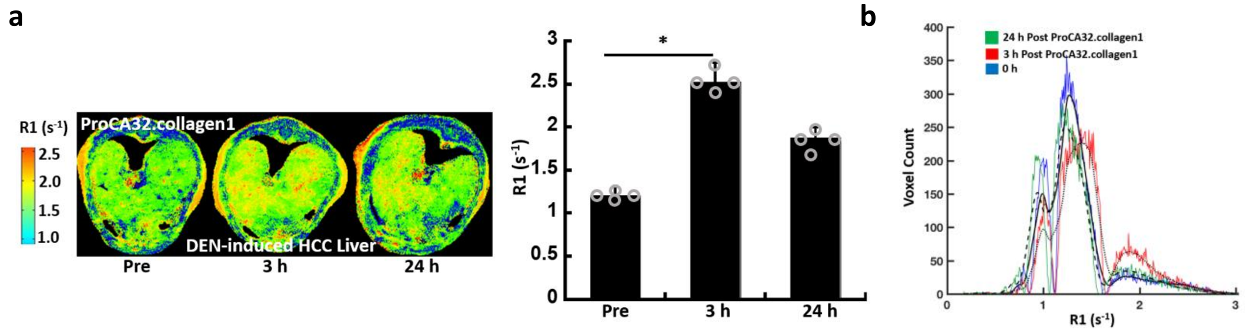

Supplementary Figure 15: Detection of collagen heterogeneity in DEN-induced mouse model. **a:** R1 map results showing collagen heterogeneity in DEN-induced diseased liver 3 and 24 hours post injection of ProCA32.collagen1. Quantitative analysis of R1 values showed an increase in 3 h post-injection as well as collagen heterogeneity in different areas. **b:** Histogram analysis of DEN-induced diseased liver pre (blue), 3 h (red) and 24 h (green) post-injection of ProCA32.collagen1. *$P < 0.05$, unpaired two-tailed student's t-test; Data are represented as mean ± SD (n=4).

Supplementary Figure 16

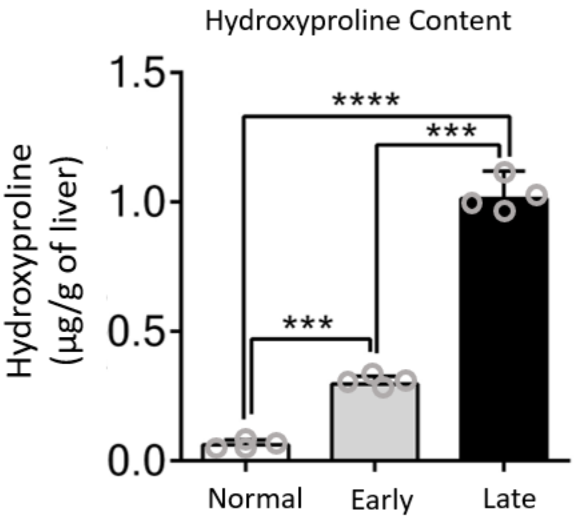

Supplementary Figure 16: Measurement of hydroxyproline content in normal liver (Ishak stage 0 of 6), early-stage (Ishak stage 3 of 6) and late-stage liver fibrosis (Ishak stage 5 of 6) in TAA/Alcohol model. ***P < 0.001, ****P < 0.0001, unpaired two-tailed student's t-test; Data are represented as mean ± SD (n=4).

Supplementary Figure 17

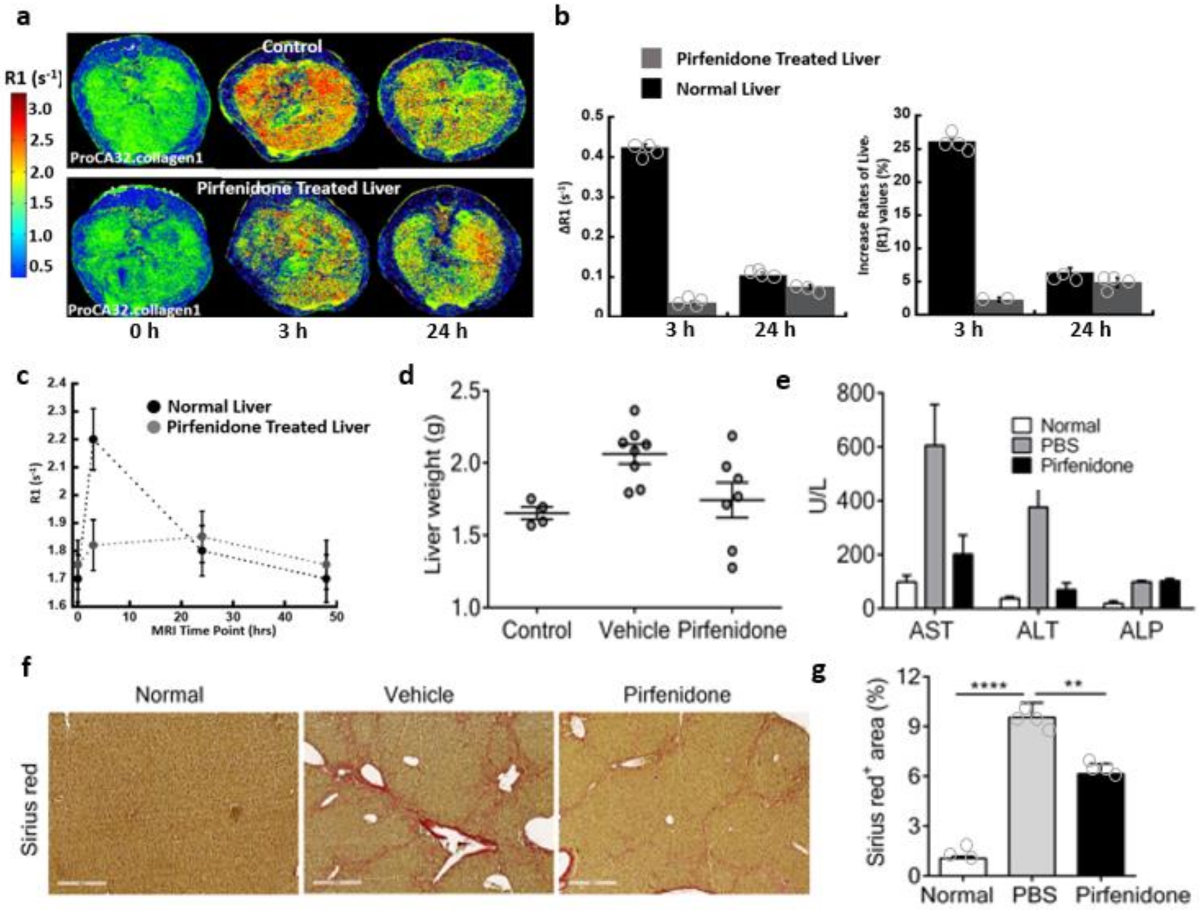

Supplementary Figure 17: Liver fibrosis treatment monitoring with ProCA32.collagen1. **a:** R1 map images of normal vs Pirfenidone treated liver before and 3 and 24 h post injection of ProCA32.collagen1. **b:** Quantitative analysis of ΔR1 and percentage of R1 increase rate 3 and 24 h post injection of ProCA32.collagen1 compared to Prescan. **c:**R1 changes of normal and drug treated liver over different MRI time points after injection of ProCA32.collagen1. **d:** Liver weights of the animals at end point of experiments and at end of fibrosis induction or after Pirfenidone treatment or buffer treatment. **e:** Liver enzyme levels after treatments with buffer (PBS), normal liver and Pirfenidone treated liver. **f:** Sirius red stains of livers treated in different conditions. **g:** Percentage of Sirius red stained areas in livers treated with buffer (PBS, cirrhosis), normal liver and Pirfenidone, scale bar, 300 µm; ** $P < 0.05$, **** $P < 0.0001$, unpaired two-tailed student's t-test; Data are represented as mean ± SD (n=4).
