# Supplementary material for: Early detection and staging of chronic liver diseases with a protein MRI contrast agent
Source: Nat Commun. 2019 Oct 29;10:4777. doi: 10.1038/s41467-019-11984-2 (PMC6820552; doi:10.1038/s41467-019-11984-2)
Supplement: Supplementary file 3 — Reporting Summary [file 41467_2019_11984_MOESM3_ESM.pdf]

nature research

Corresponding author(s): NCOMMS-18-29109A-Z

Reporting Summary

Nature Research wishes to improve the reproducibility of the work that we publish. This form provides structure for consistency and transparency in reporting. For further information on Nature Research policies, see Authors & Referees and the Editorial Policy Checklist.

Statistical parameters

When statistical analyses are reported, confirm that the following items are present in the relevant location (e.g. figure legend, table legend, main text, or Methods section).

n/a	Confirmed	
☐	☒	The exact sample size (*n*) for each experimental group/condition, given as a discrete number and unit of measurement
☐	☒	An indication of whether measurements were taken from distinct samples or whether the same sample was measured repeatedly
☐	☒	The statistical test(s) used AND whether they are one- or two-sided<br>*Only common tests should be described solely by name; describe more complex techniques in the Methods section.*
☒	☐	A description of all covariates tested
☐	☒	A description of any assumptions or corrections, such as tests of normality and adjustment for multiple comparisons
☐	☒	A full description of the statistics including central tendency (e.g. means) or other basic estimates (e.g. regression coefficient) AND variation (e.g. standard deviation) or associated estimates of uncertainty (e.g. confidence intervals)
☐	☒	For null hypothesis testing, the test statistic (e.g. *F*, *t*, *r*) with confidence intervals, effect sizes, degrees of freedom and *P* value noted<br>*Give P values as exact values whenever suitable.*
☒	☐	For Bayesian analysis, information on the choice of priors and Markov chain Monte Carlo settings
☒	☐	For hierarchical and complex designs, identification of the appropriate level for tests and full reporting of outcomes
☒	☐	Estimates of effect sizes (e.g. Cohen's *d*, Pearson's *r*), indicating how they were calculated
☐	☒	Clearly defined error bars<br>*State explicitly what error bars represent (e.g. SD, SE, CI)*

Our web collection on statistics for biologists may be useful.

Software and code

Policy information about availability of computer code

Data collection

MATLAB, MRIcron and ImgaeJ softwares were used to process MRI data. Custom codes in MATLAB were used to process histogram data (Fig. 3 h) and analyze MR images in Fig. 4f and g. Aedes MATLAB toolbox was used to process T1 map inversion recovery MR images, and ImageJ was used to proces T2 map data as well as the rest of the MRI data.
The following code was used to generate histograms of T1 maps or R1 maps, also it
can be used for a single slice/multi slice or multi scans. Based on the demand line 9 and 10 must be modified:

```
[filename,pathname] = uigetfile('*.nii','Select the *.nii image file');
  x = load_untouch_nii(fullfile(pathname,filename));
      img = x.img;
          T1=mean(img,3);
             R1=T1.^-1;
for i=1:256;
     for j=1:256;
         if R1(i,j)<4 & R1(i,j)>0;
              R11(i,j)=R1(i,j);
             else R11(i,j)=NaN;
         end;
      end;
   end;
```

```
% imagesc(R(:,:)');axis image; colormap jet;
 R12=R11;

 [filename,pathname] = uigetfile('*.nii','Select the *.nii image file');
   x = load_untouch_nii(fullfile(pathname,filename));
        img = x.img;
             T1=mean(img,3);
                R1=T1.^-1;
for i=1:256;
      for j=1:256;
          if R1(i,j)<4 & R1(i,j)>0;
                R11(i,j)=R1(i,j);
               else R11(i,j)=NaN;
          end;
       end;
    end;

 R13=R11;

 [filename,pathname] = uigetfile('*.nii','Select the *.nii image file');
   x = load_untouch_nii(fullfile(pathname,filename));
        img = x.img;
             T1=mean(img,3);
                R1=T1.^-1;
for i=1:256;
      for j=1:256;
          if R1(i,j)<4 & R1(i,j)>0;
                R11(i,j)=R1(i,j);
               else R11(i,j)=NaN;
          end;
       end;
    end;

 R14=R11;

 % in this section we will add the a tred line to the histograms
            nbins =750;
 edges_0 = linspace(min(R12(~isnan(R12))),max(R12(~isnan(R12))),nbins);
                  [N12,edges] = histcounts(R12,edges_0);
             N12(1,nbins)=0;
           N112=conv(N12,ones(1,25),'same')/25;

 edges_0 = linspace(min(R13(~isnan(R13))),max(R13(~isnan(R13))),nbins);
                  [N13,edges] = histcounts(R13,edges_0);
             N13(1,nbins)=0;
           N113=conv(N13,ones(1,25),'same')/25;

 edges_0 = linspace(min(R14(~isnan(R14))),max(R14(~isnan(R14))),nbins);
                  [N14,edges] = histcounts(R14,edges_0);
             N14(1,nbins)=0;
           N114=conv(N14,ones(1,25),'same')/25;

   % in this section we plot the histograms

   plot (edges_0,N12,'color',[100/255 149/255 237/255]);
     hold on;
       plot (edges_0, N112,'LineWidth',2,'linestyle','-','color','k');

   plot (edges_0,N13,'color',[178/255 34/255 34/255]);
       plot (edges_0, N113,'LineWidth',2,'linestyle',':','color','k');

   plot (edges_0,N14,'color', [.0 .6 .2]);
       plot (edges_0, N114,'LineWidth',2,'linestyle','--','color','k');
           hold off;
```

For Figures 4f and 4g to generate multicolor maps using MATLAB the following custom code was used:

```
[filename,pathname] = uigetfile('*.nii','Select the *.nii image file');
    x = load_untouch_nii(fullfile(pathname,filename));
        img = x.img;
             R1=img;
  for i=1:size(R1,1);
      for j=1:size(R1,2);
          if  R1(i,j)>0;
```

```
            R(i,j)=R1(i,j);
            else R(i,j)=1;
        end;
      end;
    end;

  R01=R;

  [filename,pathname] = uigetfile('*.nii','Select the *.nii image file');
    x = load_untouch_nii(fullfile(pathname,filename));
       img = x.img;
           R1=img;
    for i=1:size(R1,1);
      for j=1:size(R1,2);
        if  R1(i,j)>0;
            R(i,j)=R1(i,j);
            else R(i,j)=nan;
        end;
      end;
    end;
  R11=R;

  [filename,pathname] = uigetfile('*.nii','Select the *.nii image file');
    x = load_untouch_nii(fullfile(pathname,filename));
       img = x.img;
           R1=img;

   for i=1:size(R1,1);
      for j=1:size(R1,2);
        if  R1(i,j)>0;
            R(i,j)=R1(i,j);
            else R(i,j)=nan;
        end;
      end;
    end;

  R21=R;

%% Calculate the enhancements
%% Absolout Gain (How much enhancement do we have?)
        G1=(R11-R01)./R01;
          g1=100*G1;g1(g1<0)=nan;g1(g1>1000)=nan;
            G2=(R21-R01)./R01;
              g2=100*G2;g2(g2<0)=nan;g2(g2>1000)=nan;
% Absolout Maintain (How much washout do we have?)
          mT=g2-g1;
            m=mT; m(m<0)=nan;
              w=mT; w(w>0)=nan;

%% Binning
% %Create the un equal bins
 edges_0 = [0 10 20 30 40 50 60 70 80 90 100 1000];
                [N11,edges] = histcounts(g1,edges_0);

 edges_0 =  [0 10 20 30 40 50 60 70 80 90 100 1000]
                [N12,edges] = histcounts(g2,edges_0);

 edges_0 = [0 10 20 30 40 50 60 70 80 90 100 1000]
                [N13,edges] = histcounts(m,edges_0);

 edges_0 = [-1000 -100 -90 -80 -70 -60 -50 -40 -30 -20 -10 0];
                [N15,edges] = histcounts(w,edges_0);
               N14=fliplr(N15);
        TT=[N11' N12' N13' N14'];

%Create the equal bins
%
% nbins=10;
%  edges_0 = linspace(min(g1(~isnan(g1))),max(g1(~isnan(g1))),nbins);
%                  [N11,edges] = histcounts(g1,edges_0);
%
% edges_0 = linspace(min(g2(~isnan(g2))),max(g2(~isnan(g2))),nbins);
%                  [N12,edges] = histcounts(g2,edges_0);
%
%  edges_0 = linspace(min(m(~isnan(m))),max(m(~isnan(m))),nbins);
```

```
%                  [N13,edges] = histcounts(m,edges_0);
%
%  edges_0 = linspace(min(w(~isnan(w))),max(w(~isnan(w))),nbins);
%                  [N15,edges] = histcounts(w,edges_0);
%                  N14=fliplr(N15);
%            TT=[N11' N12' N13' N14'];
%
            %% Plot the figures
%% Figure 1 shows the initial state
folder = '/Users/Maysam/Documents/Maps/T2W' %Folder to save figures
figure;
   imagesc(R01(:,:)');axis image; colormap parula; colorbar('off');
      title('Initial'); filename='Initial';
         saveas(gca, fullfile(folder, filename), 'png');
   %Figure 2 shows the state 1
   figure;
      imagesc(R11(:,:)');axis image; colormap parula; colorbar('off');
         title('After 3 Hrs');filename='3Hrs';
            saveas(gca, fullfile(folder, filename), 'png');
      %Figure 3 shows the state 2
      figure;
         imagesc(R21(:,:)');axis image; colormap parula;colorbar('off');
            title('After 24 Hrs');filename='24Hrs';
               saveas(gca, fullfile(folder, filename), 'png');

folder = '/Users/Maysam/Documents/Maps/T2W' %Folder to save figures
%Figure 4 shows Absolout enhancement after 3 hrs
figure;
   imagesc(g1(:,:)');axis image; colormap cool;colorbar('off');
      title('Absolout enhancement after 3 hrs');filename='ABs_En_3Hrs';
         saveas(gca, fullfile(folder, filename), 'png');
% figure;
%     imagesc(N11(:,:));axis image; colormap cool;colorbar('off');
%        title('Distribution Absolout enhancement after 3 hrs');filename='DABs_En_3Hrs';
%           saveas(gca, fullfile(folder, filename), 'png');

   %Figure 5 Absolout enhancement after 24 hrs
   figure;
      imagesc(g2(:,:)');axis image; colormap hot;colorbar('off');
         title('Absolout enhancement after 24 hrs');filename='ABs_En_24Hrs';
            saveas(gca, fullfile(folder, filename), 'png');
%     figure;
%        imagesc(N12(:,:));axis image; colormap hot;colorbar('off');
%           title('Distribution Absolout enhancement after 24 hrs');filename='DABs_En_24Hrs';
%              saveas(gca, fullfile(folder, filename), 'png');
      %Figure 6 Maintained vs Washout
      figure ;
         imagesc(mT(:,:)');axis image; colormap jet;colorbar('off');
            title('Maintained vs Washout');filename='MvW';
               saveas(gca, fullfile(folder, filename), 'png');

         %Figure 7 Maintained
         figure ;
            imagesc(m(:,:)');axis image; colormap hot;colorbar('off');
               title('Maintained');filename='Main';
                  saveas(gca, fullfile(folder, filename), 'png');
%           figure ;
%              imagesc(N13(:,:));axis image; colormap hot;colorbar('off');
%                 title('Distribution Maintained');filename='DMain';
%                    saveas(gca, fullfile(folder, filename), 'png');

            %Figure 8 Washout
            figure;
               imagesc(w(:,:)');axis image; colormap cool;colorbar('off');
                  title('Washout');filename='Wash';
                     saveas(gca, fullfile(folder, filename), 'png');
%              figure;
%                 imagesc(N14(:,:)');axis image; colormap cool;colorbar('off');
%                    title('Distribution Washout');filename='DWash';
%                       saveas(gca, fullfile(folder, filename), 'png');

figure;
   imagesc(TT(:,:));axis image; colormap jet;colorbar;
      set (gca,'YDir','normal');
         namesx = {'3 Hours'; '24 Hours'; 'Maintained'; 'Washout'};
```

```
set(gca,'xtick',[1:4],'xticklabel',namesx);
  namesy = {'%10<'; '%10-%20'; '%20-%30'; '%30-%40'; '%40-%50'; '%50-%60'; '%60-%70'; '%70-%80'; '%80-%90'; '%90-%100'; '%100<'};
    set(gca,'ytick',[1:11],'yticklabel',namesy);
      title('T2W Quantitative');filename='TT2WQ';
        saveas(gca, fullfile(folder, filename), 'png');
```

WinNonlin ™ 5.0.1 was used for analysis of pharmacokinetics data. R and SAS softwares were used for ROC and AUC analysis were used for statistical analysis. GraphPad Prism 5 was used for CNR, ΔR1 and ΔR2 statistical calculations.

Data analysis	MATLAB 2016 (Mathworks, Natick, MA) with academic license. ImageJ 1.51j8 which is publicly accessible (https://imagej.nih.gov/ij/download.html, NIH). MRIcron version 1 which is publicly accessible (http://people.cas.sc.edu/rorden/mricron/install.html). WinNonlin ™ 5.0.1. GraphPad Prism 5. R version 3.2.2 and SAS version 9.4.

For manuscripts utilizing custom algorithms or software that are central to the research but not yet described in published literature, software must be made available to editors/reviewers upon request. We strongly encourage code deposition in a community repository (e.g. GitHub). See the Nature Research guidelines for submitting code & software for further information.

Data

Policy information about availability of data

All manuscripts must include a data availability statement. This statement should provide the following information, where applicable:

- Accession codes, unique identifiers, or web links for publicly available datasets
- A list of figures that have associated raw data
- A description of any restrictions on data availability

The authors declare that all data supporting the findings of this study are available within the main text and supplementary information section.

Field-specific reporting

Please select the best fit for your research. If you are not sure, read the appropriate sections before making your selection.

☒ Life sciences ☐ Behavioural & social sciences ☐ Ecological, evolutionary & environmental sciences

For a reference copy of the document with all sections, see nature.com/authors/policies/ReportingSummary-flat.pdf

Life sciences study design

All studies must disclose on these points even when the disclosure is negative.

Sample size	Sample sizes were calculated using nQUERY assuming a two-sided alpha-level of 0.05, 80% power, and homogeneous variances for the 2 samples to be compared, with the means and common standard deviation for different parameters predicted from published data.
Data exclusions	None
Replication	For each series of experiments, all replication attempts were successful.
Randomization	In "Methods" section, In "DEN-induced liver cirrhosis" model generation and "TAA/alcohol-induced liver fibrosis and cirrhosis" model generation, all animals were randomly assigned to the groups and were included in the study. In "NASH diet-induced liver fibrosis and cirrhosis" section, in order to develop cirrhosis, liver-specific Comparative Gene Identification-58 (CGI-58) knocked out (LivKO) mice were assigned to one specific group treated with NASH diet (Guo et al., J Lip Res. 2013).
Blinding	The operators responsible for statistical data analysis were blinded and unaware of group allocation throughout the experiments. In "Methods" section, "Histology analysis", an experienced pathologist was blinded to the groups of H&E staining to evaluate the organ toxicity of ProCA32.collagen1. Furthermore, the pathologist was blinded to the groups of Sirius red and H&E staining in order to evaluate the stage of fibrosis.

Reporting for specific materials, systems and methods

Materials & experimental systems

n/a	Involved in the study
☒	☐ Unique biological materials
☐	☒ Antibodies
☒	☐ Eukaryotic cell lines
☒	☐ Palaeontology
☐	☒ Animals and other organisms
☐	☒ Human research participants

Methods

n/a	Involved in the study
☒	☐ ChIP-seq
☒	☐ Flow cytometry
☒	☐ MRI-based neuroimaging

Antibodies

Antibodies used	For immunofluorescence analysis in mice liver tissue samples, we used the following antibodies:<br>For ProCA32.collagen1 - self-generated rabbit antibody, anti-ProCA32.collagen1 prepared by Division of Animal Resources at Georgia State University (1:100 dilution) was used as primary antibody.<br>Another primary antibody for collagen type I, Anti-Collagen I antibody [COL-1] (ab34710) was used with 1:500 dilution.<br>Alexa Fluor 488-labeled goat anti-rabbit IgG (Invitrogen; catalog #R37116) was used as a secondary antibody for collagen type I with 1:200 dilution.<br>Alexa Fluor 594-labeled goat anti-rabbit IgG (Invitrogen; catalog #A-11012) was used as a secondary antibody for ProCA32.collagen1 (1:200 dilution).<br>The nuclei of the cells were stained by DAPI (blue, ThermoFisher scientific, catalog #62248, 1:1000 dilution).
Validation	The antibodies purchased have already been validated by the manufacturers. ProCA32.collagen1 antibody has been validated by previous publications.

Animals and other organisms

Policy information about studies involving animals; ARRIVE guidelines recommended for reporting animal research

Laboratory animals	In "Mouse model for development of TAA/alcohol-induced liver fibrosis and cirrhosis" section, 6-7 week old male BALB/c mice were used. In "Mouse model for development of DEN-induced liver cirrhosis" section, 14 day old male C57BL/6 mice were used. In "Mouse model for development of NASH diet-induced liver fibrosis and cirrhosis" section, 6 week old male C57BL/6 mice were used. For tissue toxicity assessment, 10 week old female CD-1 mice were used.
Wild animals	Study did not involve wild animals.
Field-collected samples	N/A

Human research participants

Policy information about studies involving human research participants

Population characteristics	Human Paraffin Embedded Tissue Array (Human HCC and normal precut liver tissue microarray) containing 20 cases of liver tumor with 4 normal tissues from autopsy, duplicated cores per case were purchased from US Biomax, Inc., catalog # LV482 and used to stain for collagen and ProCA32.collagen1. The tissue samples were collected with the donor being informed completely and with their consent. The study has been determined by IRB of Georgia State University to be exempt from federal regulations and it was determined that it meets the organization's ethical standards.<br>For serum stability experiments, human serum (sterile-filtered, human male AB plasma) was purchased from MilliporeSigma, catalog # H4522. The serum samples were collected with the donor being informed completely and with their consent. The study has been determined by the Institutional Review Board (IRB) of Georgia State University to be exempt from federal regulations and it was determined that it meets the organization's ethical standards.
Recruitment	*Describe how participants were recruited. Outline any potential self-selection bias or other biases that may be present and how these are likely to impact results.*
